# Research Progress of Biomarkers for Sepsis and Precision Medicine

**DOI:** 10.1155/emmi/4585495

**Published:** 2025-07-07

**Authors:** Neng Wang, Hansheng Huang, Youlin Tan, Nai Zhang

**Affiliations:** ^1^Graduate School, Jiangxi University of Chinese Medicine, Nanchang, China; ^2^Department of Neurology, Jiangxi Province Hospital of Integrated Chinese and Western Medicine, Nanchang, China; ^3^Department of Emergency, Jiangxi Province Hospital of Integrated Chinese and Western Medicine, Nanchang, China

**Keywords:** biomarkers, diagnosis, precision medicine, prognosis, sepsis

## Abstract

Since 1989, the definition of sepsis has been revised several times. The 2016 Sepsis-3 consensus definition of sepsis aims to improve diagnostic accuracy and reduce the frequency of misdiagnosis. The Sequential Organ Failure Assessment (SOFA) scoring system facilitates personalized treatment. Sepsis-related biomarkers are essential for diagnosis, treatment, and prognosis; however, their widespread application is limited by their insufficient sensitivity and specificity. From October 2019 to October 2024, 4801 studies had reported on sepsis-related biomarkers. The number of studies initially increased but subsequently decreased over time (beginning from 2021). C-reactive protein (CRP) and procalcitonin (PCT) are the most frequently investigated biomarkers, and their combination with other biomarkers can improve diagnostic accuracy. Advancements in data-driven technologies have helped optimize the definition of sepsis, accelerate early diagnosis, clarify subphenotypes, improve prognostic assessment, and develop personalized treatment strategies. With the deepening of research on the pathological mechanisms of sepsis, novel biomarkers such as vascular endothelin, vasoactive peptides, BMP9, cytokines, chemokines, and cfDNA have emerged, which are closely related to the severity of the disease. The clinical application of known biomarkers has expanded, and their kinetic changes are considered more accurate than a single value in predicting outcomes. In addition, related studies have focused on the exploration of precision medicine for sepsis. Efforts have been made to divide patients into more homogeneous subgroups by distinguishing their shared biological characteristics, thus providing valuable avenues for developing novel therapeutic approaches. This article reviews the research status of some commonly used sepsis biomarkers from October 2019 to October 2024, analyzes the current application status and limitations, pays attention to the changes of biomarkers and the exploration of precision medicine for sepsis, and aims to develop new treatment approaches by dividing patients into subgroups.

## 1. Introduction

Sepsis is extremely common among hospitalized patients in the intensive care unit (ICU). Sepsis not only has a high mortality rate but can also lead to long-term disability [1, 2]. Its complexity stems from the host's abnormal response to infection, which is often closely associated with acute organ dysfunction and is associated with a high risk of death, and is therefore one of the important causes of death in critically ill patients [3, 4]. Early recognition of sepsis is essential for prompt and effective treatment [5]; however, early sepsis in patients with suspected infection is often overlooked in clinical practice, leading to delayed treatment and increased risk of death [6]. The emergency department is an important setting for the evaluation of patients with suspected infection and the early signs of sepsis, but the time-critical challenge of managing patients requires rapid treatment and management decisions, and accurate identification of patients at high risk of mortality is critical not only to facilitate timely initiation of antibiotic therapy but also to guide patients on the need for further hospitalization for high-level care [7].

The incidence of sepsis has been on the rise worldwide over the past 5 years [8]. On the one hand, the development of diagnostic technology has improved the awareness and diagnostic ability of medical staff to sepsis, and on the other hand, the increase in the number of patients with underlying diseases, the aging of the population, and the increase in the frequency of invasive medical procedures are all associated with the increased risk of sepsis. The incidence of sepsis is high but relatively stable in Europe and the United States, with a high proportion of patients with sepsis in the ICU in the United States, with an incidence of about 200–300 cases per 100,000 people [9]. Some less developed regions in Africa and Asia have higher morbidity rates of 500–600 cases per 100,000 people due to poor sanitation, lack of medical resources, and low vaccine coverage. Sepsis mortality rates are about 20%–30% globally, with more than 50% in some under-resourced areas [10]. In the past 5 years, despite continuous advances in medical technology, the overall mortality rate of sepsis has not decreased significantly, and the mortality rate of patients with early sepsis is relatively low if treated promptly and effectively, but the mortality rate rises sharply to 40%–60% if it progresses to severe sepsis or septic shock [11].

The definition of sepsis has evolved several times over time, from the initial definition in 1989 to the consensus definition in 1992 to the Third International Consensus definition of Sepsis and Septic Shock (Sepsis-3) in 2016 [12]; the core goal of this evolution is to optimize diagnosis to more accurately identify features of sepsis and minimize misdiagnosis and missed diagnosis, with an emphasis on organ dysfunction having far-reaching implications for the development of more effective treatment strategies [13]. The Sequential Organ Failure Assessment (SOFA) scoring system in the Sepsis-3 definition has significantly improved in specificity and sensitivity compared to the previous criteria: the early definition uses nonspecific symptoms such as fever and tachycardia, which can easily lead to misdiagnosis of nonsepsis cases such as autoimmune diseases. The SOFA score assesses multiple organ systems (e.g., respiratory oxygenation and renal creatinine levels) through objective parameters, which can improve specificity by accurately identifying sepsis-related organ dysfunction and improving sensitivity by detecting subtle changes in early organ function through continuous monitoring, so as to make more accurate diagnosis and severity assessment, and provide a basis for individualized treatment [14]. In addition, the continuous revision of the definition has promoted the development of related scientific fields, prompting researchers to further explore the pathogenesis and targeted therapy of sepsis, especially focusing on the search for biomarkers that can accurately predict the occurrence and mortality of sepsis [15].

Although more than 100 clinical trials have been reported, effective treatments for sepsis are lacking at present. Attempts have been made to establish precision medicine approaches to address the heterogeneity of sepsis. For instance, immunotherapy has been used to treat sepsis based on precise biomarkers and molecular mechanisms that define specific immune endotypes. Risk stratification can facilitate the development of immunotherapy-based approaches, making immunotherapy a key strategy for the successful treatment of sepsis. The clinical translation of these approaches will require their comprehensive evaluation in prospective clinical studies, which may require novel adaptive trial designs. Some of these studies are currently underway; however, many challenges remain unaddressed. Categorizing patients based on clinical syndromes requires significant patient participation. Therefore, a long-term global collaboration among researchers, clinicians, industry experts, and patients is necessary for the clinical translation of precision medicine approaches and for advancements in other areas of medicine [16].

## 2. Methods

The PubMed platform was utilized to retrieve data from the Medline database from October 2019 to October 2024. In this study, we used a search strategy that comprehensively covered relevant literature, and the core search terms included “biomarkers” and “sepsis” and “precision medicine” and “sepsis.” For example, when searching for biomarker-related studies, the specific search query is “biomarker [Title/Abstract] AND sepsis [Title/Abstract]” to ensure that the search documents directly relate to the association of biomarkers with sepsis in the title or abstract.

Additional keywords related to specific biomarkers, such as “CRP [Title/Abstract] AND biomarker [Title/Abstract] AND sepsis [Title/Abstract]” were used to further refine the search, in order to obtain in-depth studies of individual biomarkers.

This study did not use medical subject terms (MESH) as the primary search strategy, because the selected keywords are more directly related to sepsis biomarkers and emerging specific research topics in the field of precision medicine, and the use of MESH terminology may not only omit some of the latest professional studies that are not fully included in the preset categories but also acknowledges the value of MESH terminology in a broader and comprehensive search of biomedical literature.

This review excluded studies that did not focus on sepsis-related biomarkers or sepsis precision medicine and excluded nonoriginal literature such as case reports, reviews, and editorials, as well as studies with incomplete biomarker testing data or precision medicine interventions, to ensure that the review included only high-quality relevant studies that more accurately reflected the current state of knowledge in the field.

This review assessed the quality of the study according to the PRISMA guidelines: in terms of study design, randomized controlled trials were preferred over observational studies due to their higher internal validity; an adequate sample size ensures statistical validity, and reasonable sampling reduces selection bias. Biomarker testing methods and precision medicine interventions are also carefully reviewed, with validated biomarker measurement techniques and clearly described intervention processes being important quality indicators. Finally, the results of the evaluation report are considered to be of higher quality as having clear data presentation, comprehensive statistical analysis, and reasonable interpretation of the findings can help critically evaluate the research and draw reliable conclusions.

Detailed search syntax, exclusion reasons, and quality assessment tools are provided in Supporting Information (available here).

## 3. Results

**Figure 1:**
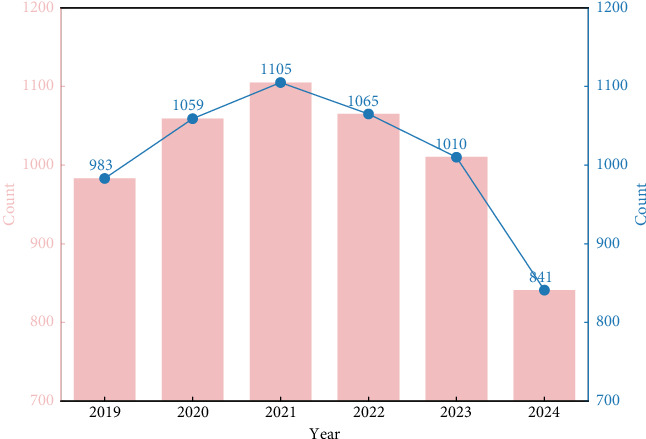
Number of relevant studies on sepsis-related biomarkers during the past 5 years.

Between October 2019 and October 2024, over 250 sepsis-related biomarkers were identified and evaluated, and 4801 studies meeting the inclusion criteria were published. The number of biomarker-related studies initially rose but started to decline annually after 2021 (Figure 1).

The early increase in sepsis-related biomarker studies was driven by multiple factors. Given sepsis' high mortality and complexity, the Sepsis-3 definition in 2016, with its focus on organ dysfunction and the SOFA scoring system, offered a fresh research angle, prompting researchers to explore biomarkers linked to organ function and prognosis. Technological progress in laboratory detection, such as improved immunoassay and molecular biology methods, also made biomarker identification and measurement more precise, facilitating in-depth research on C-reactive protein (CRP), procalcitonin (PCT), and interleukin-6 (IL-6) [17].

However, the post-2021 decline can be attributed to research hotspot saturation. Traditional biomarkers such as CRP and PCT had been thoroughly studied, making it hard to obtain novel and significant results. Moreover, some research directions faced bottlenecks. Novel biomarkers often had issues such as instability, complex detection, and limited clinical validation, hampering their translation into clinical use [18].

CRP and PCT were the most-studied biomarkers, followed by IL-6, presepsin, and CD64. Data-driven technologies have advanced sepsis research, optimizing its definition, speeding up early diagnosis, identifying subphenotypes, improving prognosis assessment, and enabling personalized treatment. A relevant study used vital sign trajectories to identify and validate a new subphenotype of sepsis; a total of 20,729 patients with suspected infection were included and four subtypes of sepsis were identified, and there were differences in age, comorbidities, medication use, mortality risk, and laboratory indicators among each subphenotype, but the addition of laboratory indicators had little effect on the classification, and Groups A and D still had a higher 30 day mortality rate after adjusting for confounding factors. In the SMART trial, 834 patients with sepsis were classified into these 4 subphenotypes, with patients in Group D treated with balanced crystalloid having lower mortality and all subphenotypes being associated with treatment allocation. However, despite many biomarkers, there is still no clear definition of abnormal immune responses in sepsis and no widely-accepted diagnostic tool [19]. More than 250 sepsis-related biomarkers were evaluated in the 4801 studies included between October 2019 and October 2024. There are significant differences in the diagnostic and prognostic value of traditional markers (e.g., CRP and PCT) and emerging markers (e.g., Bone morphogenetic protein 9 (BMP9) and endothelin). To compare the performance of the system, the sensitivity, specificity, and clinical characteristics of key biomarkers were collated based on the study data (Table 1).

**Figure 2:**
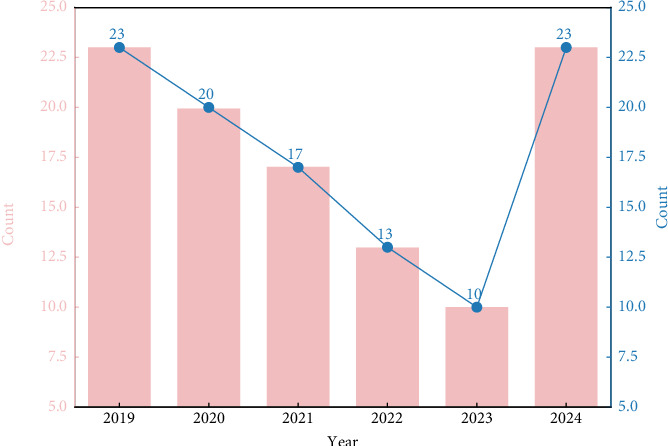
Number of relevant studies on precision medicine in sepsis during the past 5 years.

Studies on sepsis precision medicine were initially few, showing a decline in the first 4 years but an exponential growth in 2024. The early decline was due to unclear research directions and technological constraints. In the early days, the concept was new, and researchers struggled with patient classification and targeted treatment strategies [32]. The lack of high-quality data and effective analysis methods also held back progress. The 2024 growth is mainly due to technological breakthroughs. Artificial intelligence (AI) and big data technologies allow for more accurate patient subgroup identification and personalized treatment planning by analyzing diverse patient data. Machine learning algorithms, for example, can analyze the relationship between multiple factors and sepsis outcomes. In addition, increased funding from governments and research institutions worldwide has attracted more researchers, fueling the growth of sepsis precision medicine studies [33] (Figure 2). Precision medicine for sepsis aims to optimize treatment by accurately evaluating patients and adjusting drug dosages, reducing adverse reactions. It helps doctors adjust antibiotic regimens in a timely manner, optimize the overall treatment plan through therapeutic effect monitoring, and adjust ineffective treatments promptly. The evaluation of treatment effects also provides references for future treatments [34].

Specific definitions of the grading criteria for biomarker sensitivity and specificity are provided in Supporting Information (available here).

## 4. Discussion

### 4.1. Biomarkers

The incidence of sepsis defined by the Sepsis-3 criteria among suspected infection patients in the emergency department is estimated to be as high as 30%, with an in-hospital mortality rate exceeding 10%. Administration of antibiotics within the first hour of admission can effectively improve survival rates, highlighting the importance of early diagnosis. The clinical manifestations of infection are often nonspecific, and although fever is a typical sign of infection, about half of the cases may not have fever, leading to delayed treatment. Suspected sepsis patients often first visit the emergency department, and accurately identifying such patients is a major clinical challenge and leads to delays in initiating appropriate treatment. Therefore, it is particularly necessary to improve the diagnosis of bacterial infections and sepsis and patient risk stratification to assist clinical decision-making. In-depth research on biomarkers is needed to achieve these goals [35].

Biomarkers play a potential key role in achieving accurate diagnosis, and traditional biomarkers such as CRP levels, serum PCT levels, and white blood cell count (WBC) have been widely used for bacterial infection diagnosis. Although these biomarkers are widely used in clinical practice, they have some limitations in distinguishing sepsis from other inflammatory diseases or predicting disease progression. For example, in nonbacterial infection cases such as COVID-19, the level of these biomarkers has no difference from sepsis [36]. The inflammatory response activated by damage-associated molecular patterns (DAMPs) is quite similar to that produced by sepsis, and similar changes in these biomarkers may also be observed in systemic inflammatory response syndrome (SIRS) [37].

In addition to the SOFA score, which is considered the gold standard for assessing the severity of sepsis but may not be easily obtained in emergency settings, blood lactate levels are one of the most commonly used biomarkers in suspected sepsis cases. Elevated lactate levels indicate systemic tissue hypoperfusion and cellular dysfunction in sepsis patients. Blood lactate levels have been included in the definition criteria for septic shock and can be quickly detected in a short period of time [13]. The role of blood lactate in sepsis assessment seems to have weakened as this indicator is no longer included in the definition of sepsis [38].

BMP9 has significant implications in the field of medical research. A study on two sepsis patient cohorts quantified circulating BMP9 concentrations compared to healthy individuals. Sepsis patients had lower serum BMP9 concentrations upon admission and were significantly associated with 28-day mortality. Patients with a higher risk of death had lower BMP9 concentrations upon admission [19].

When sepsis occurs, the immune system activates and releases inflammatory factors such as tumor necrosis factor alpha, which activate transcription factor NF-κB in endothelial cells through signaling pathways. NF-κB promotes the transcription of endothelin, which activates multiple signaling pathways by binding to corresponding receptors [39]. This activation leads to dysfunction and damage of vascular endothelial cells, manifested as increased vascular permeability and hypercoagulability. In the early stage of sepsis, the level of endothelin is significantly elevated, which can reflect the pathological status of patients earlier than traditional indicators and can be used as a sensitive biomarker for early diagnosis of sepsis. The level of endothelin is closely related to the severity of sepsis and can be used to evaluate disease progression and prognosis. In addition, changes in endothelin levels during treatment can reflect the therapeutic effect [40]. Recent studies have elucidated the regulatory mechanism of endothelin in sepsis and its role in the diagnosis, monitoring, and prognosis of sepsis. The detection methods of endothelin have been continuously optimized, and there have been extensive developments in liquid chromatography–mass spectrometry technology and biosensors. Inflammatory factors can regulate endothelin expression through transcription factors, among which hypoxia inducible factors play a key role in regulating endothelin expression in sepsis-related vascular endothelial injury [41]. Changes in the expression and distribution of endothelin receptors in sepsis exacerbate endothelial damage and dysfunction. The association between endothelin and other sepsis-related biomarkers provides a new approach for comprehensive disease assessment [42, 43].

Compared to a single test, the dynamic changes of biomarkers such as PCT, CRP, or endothelin (such as continuous monitoring) have advantages in both theory and practice. For example, an upward trend in PCT (> 0.5 ng/mL every 24 h) indicates sustained bacterial proliferation or treatment failure, while a sustained decrease (> 30% decrease within 48 h) indicates a good response to antibiotic treatment. This dynamic monitoring method is consistent with the pathophysiological characteristics of sepsis, as the immune response and organ dysfunction of sepsis patients change over time. Compared to static values that may reflect temporary inflammation (such as transient elevation of CRP after trauma), trend analysis can help distinguish between true sepsis progression and acute phase reactions [44].

### 4.2. Challenges

Pathogen-associated molecular patterns (PAMPs) activated during sepsis trigger the release of innate immune and inflammatory mediators through the same receptors as DAMPs [45]. This commonality partly explains the difficulty of identifying specific biomarkers [46, 47]. The complexity of biomarkers is affected by the type of infection (viral infection mainly activates DAMPs, while bacterial infection mainly activates PAMPs), and the type of infection may have an impact on the expression of biomarkers [45, 48]. On the other hand, when it comes to guiding resuscitation, the performance of biomarkers is even inferior to that of some simple clinical indicators during septic shock [49]. At present, although biomarkers have potential diagnostic and therapeutic value, there is a lack of markers that can stably and reliably assist in identifying bacterial sepsis or evaluating the severity of sepsis. Sepsis, as a complex clinical condition, has diverse manifestations. A single biomarker cannot fully reflect its complex characteristics but can only be used as a specific and rapid change indicator of the pathophysiological process of sepsis. However, technological progress has brought new hope. AI shows great potential in sepsis prediction. In 2020, a study in the New England Journal of Medicine analyzed the data of more than 5000 patients with sepsis. After data preprocessing and splitting using the long-term and short-term memory network (LSTM), the AI model can predict the onset of sepsis 24 h in advance, with an accuracy rate of 88%, which is 30% higher than that of traditional methods [50]. In 2019, a study in Nature Genetics conducted genome sequencing on patients and control populations and identified five susceptibility-related genes and three severity-related genes. The sensitivity and specificity of its biomarker model reached 78% and 82%, which can realize early risk assessment. Both omics and AI play a powerful role [51]. Omics explores the mechanism of sepsis, while AI integrates biomarkers. AI can help improve the diagnosis, prognosis, evaluation, and individualized treatment level by analyzing the pathophysiological process of sepsis through big data. AI and omics have broad prospects in sepsis research but face challenges. Data privacy is the main problem. The sepsis prediction model based on AI relies on large-scale sensitive patient data. Data leakage may cause problems such as insurance or employment discrimination. Protecting such data requires strict security measures and consumes a lot of resources. Omics technology also raises privacy concerns. Genomic data have high personal attributes and can reveal a large amount of individual information [52]; the integration of omics data and clinical data complicates privacy issues, and the lack of a regulatory framework leads to difficulties in data management. Integrating AI and omics data into existing medical systems is another challenge. Medical institutions using traditional systems and integrating new technologies need to make a lot of effort in software development, data standardization, and ensuring interoperability [53], as compatibility issues can limit the practical use of these technologies.

### 4.3. Precision Medicine

Sepsis is common in critically ill patients as a systemic response to microbial infection, and although numerous attempts to develop targeted therapies for sepsis have shown heterogeneity between patients [54], the current consensus definition of sepsis encompasses a broad range of criteria, emphasizing life-threatening organ dysfunction due to the host's abnormal response to infection, and that sepsis heterogeneity is attributable to a variety of factors, including different infectious etiologies, unique comorbidities and genetic backgrounds of individual hosts, and differences in timing of diagnosis and treatment [55]; these factors not only influence the course of the disease in patients with sepsis but also determine their response to therapeutic interventions, and although there is a better understanding of the heterogeneity of the immune response to sepsis, disease management is still limited to prevention, early recognition, and supportive care [56]. Although more than 100 clinical trials have attempted to treat sepsis by modulating the systemic inflammatory response, none have been significantly successful [4, 5, 57]. Due to the heterogeneity of sepsis and the lack of effective treatment methods, researchers have turned their focus to precision medicine for the diagnosis and treatment of sepsis; a related study used vital sign trajectories to identify and validate new subphenotypes of sepsis; the study evaluated the clinical characteristics and outcomes of these subphenotypes, and there is a manuscript to introduce the SMART study to identify four sepsis subphenotypes but not to clarify their clinical translational value. Another study using a retrospective observational design for secondary analysis of SMART trial data identified and validated a neosepsis subphenotype by vital sign trajectories [58].

### 4.4. Study Design and Process

Patients with suspected infection in Emory Medical System were divided into the training group (2014–2017) and validation group (2018-2019) after excluding some cases, and after dealing with the error recording of relevant vital sign indicators, a subphenotypic model was constructed in the training group based on the group trajectory model (GBTM) and validated in the validation group, and the model performance was evaluated by subphenotypic differences, laboratory index effects, association with 30-day mortality, and intravenous infusion therapy effect.

### 4.5. Clinical Implementation

#### 4.5.1. Diagnosis

Clinicians can preliminarily classify patients based on vital signs 8 h prior to admission, such as high fever, tachycardia, tachypnea, and hypotension suggestive of Group A, and milder symptoms but more comorbidities may be Group B. Age and comorbidities also vary by subphenotype (younger in Groups A and B with fewer comorbidities; older in Groups C and D with more comorbidities), and clinical diagnosis is based on a combination of vital signs and laboratory indicators, the latter of which are used to validate or Supporting Information (available here) [59].

#### 4.5.2. Treatment

Treatment response varied among different subphenotypes, with a better prognosis in Group D patients in the SMART trial using balanced crystalloid fluid and antibiotic selection and duration tailored to subphenotype based on pathogen characteristics and immune response. The use of vasopressors was higher in Groups A and D, and the use of inotropes in Group D was higher, suggesting that rational drug use according to subphenotype is required [60].

### 4.6. Precision Medicine and Biomarker-Guided Personalized Treatment

It is important to construct a biomarker-driven individualized treatment framework in sepsis precision medicine, and this paper proposes a decision tree model to demonstrate the association between biomarker profiles and specific treatments.

#### 4.6.1. Initial Biomarker Assessment

Key sepsis biomarkers such as PCT, CRP, and IL-6 are measured at the time of patient evaluation, with a PCT > 2 ng/mL that often indicates a serious bacterial infection requiring aggressive antibiotic therapy and an elevated PCT < 0.5 ng/mL, but CRP and IL-6 indicate a complication of nonisolated bacterial inflammation and a shift to another clade [61].

#### 4.6.2. Antibiotic Therapy Decision

Previous antibiotic exposure and local resistance should be considered for clades with high PCT levels, and broad-spectrum β-lactam antibiotics are recommended in the absence of recent exposure and high β-lactam susceptibility, and alternatives such as carbapenems or vancomycin are recommended based on the suspected pathogen in the presence of exposure or resistance [62, 63].

#### 4.6.3. Immunomodulatory Therapy Consideration

Branches with high CRP and IL-6 but normal PCT should be evaluated for immune-related biomarkers such as lymphocyte subsets (CD4+ and CD8+ T cells) and natural killer cell activity, immunomodulatory regimens such as thymopeptide α-1 may be considered for immunosuppression with low CD4+ T cell counts, and other immunomodulatory therapies may be initiated if natural killer cell activity is low [64, 65].

#### 4.6.4. Monitoring and Treatment Adjustment

The decision tree contains nodes for continuous monitoring of biomarkers and the absence of a decrease in PCT after 48 h of antibiotic therapy may indicate treatment failure or resistance, requiring re-evaluation of antibiotic selection, such as blood cultures, and non-normalization of CRP and IL-6 after immunomodulatory therapy, which may require adjustment or supplementation of interventions [66].

The decision tree model provides a practical path for the individualized treatment of sepsis using biomarker profiles, strengthens the discussion of precision medicine, and guides clinicians.

### 4.7. Limitations of the Subphenotyping Study

There are methodological limitations in subphenotypic studies: retrospective design based on secondary analysis of SMART trial data may introduce inherent bias; reliance on existing data may be incomplete, inaccurate, or lead to selection bias; data collection at different time periods and medical institutions lacks standardization; confounding factors affect the identification and characterization of subphenotypes of sepsis; and the association of subphenotypes with clinical outcomes such as mortality and treatment response may be overestimated or underestimated [67].

Another significant limitation is the potential inadequacy of sample size, although the study included 20,729 patients with suspected infection, the sample size of each subgroup may be small after further subtyping, resulting in reduced statistical power, difficulty in detecting subtle differences between subphenotypes, increased uncertainty in estimating effects, and difficulty in generalizing the results to a broader population of patients with sepsis, limiting the clinical applicability of the subphenotypic model [68].

These limitations affect the interpretation and application of subphenotypic models: the bias of retrospective design and the lack of statistical power due to small samples weaken the reliability of model predictions and treatment recommendations, and clinicians may be reluctant to make treatment decisions based solely on this model; The lack of generalizability means that the model may not be applicable to all patients with sepsis, especially those of different ethnicities, regions, or comorbidities [69].

Taken together, the entire discussion on the diagnostic and prognostic value of biomarkers, as well as the exploration of precision medicine for sepsis, is firmly based on 4801 articles retrieved from PubMed. These articles have been carefully screened and analyzed to distill existing knowledge and emerging trends in this complex and critical area of medical research.

Future studies need to focus on prospective studies that include larger and more diverse patient populations, reduce the risk of bias through standardized data collection protocols, expand sample sizes to improve statistical power, and achieve more accurate subphenotype identification and association analysis with clinical outcomes. Multicenter studies can be carried out to cover medical institutions in different regions; increase the sample size and include sepsis patients in various medical scenarios; and integrate more comprehensive data, such as genetic information, microbiome data, and real-time physiological monitoring, to improve the accuracy of subphenotypic segmentation and deepen understanding of sepsis heterogeneity [58].

## 5. Conclusion

Sepsis has high morbidity and mortality, and early diagnosis is critical but difficult due to the lack of specificity of symptoms. The specificity of traditional biomarkers (CRP, PCT, and WBC) is insufficient, and new markers (BMP9 and endothelin) are affected by factors such as underlying diseases, and the complexity of the immune response also increases the difficulty of screening. In the future, omics and AI may help develop precise diagnostic tools by analyzing molecular mechanisms and mining data. The heterogeneity of sepsis leads to limited traditional treatment, and the integration of data can assist in identifying subphenotypes and formulating differentiated regimens, but the model based on retrospective studies is not universal, and multipopulation validation and more data need to be included. Cross-industry collaboration (targeted drug development and diagnostic tool improvement) and interdisciplinary research (wearable device monitoring and AI classification) are necessary, and international alliances need to focus on regional differences for validation. Although there has been progress in related research, effective responses still need to be explored.

## Figures and Tables

**Table 1:** Sensitivity, specificity, and clinical utility of biomarkers in sepsis.

Biomarker	Sensitivity	Specificity	Clinical utility in sepsis	Limitations
CRP	Moderate (markedly elevated in bacterial infection, 50%–80%) [20]	Low (< 50%, noninfectious inflammation such as SIRS, may be elevated on trauma) [21]	It is often used in the initial screening of infection to assist in determining the degree of inflammatory activity; ambulatory monitoring assesses response to treatment [22]	Nonsepsis-specific; it is affected by factors such as age, liver and kidney function, and hormone therapy [23]
PCT	Moderate (markedly elevated in bacterial sepsis, 50%–80%) [24]	Medium (50%–80%, more specific for bacterial infection than CRP) [25]	Distinguish between bacterial and nonbacterial infections; length of antibiotic use (e.g., a 30% drop in procalcitonin > indicates response to treatment) [26]	May be normal in viral infection, but may be mildly elevated in severe stress (e.g., major surgery); neonates and patients with renal insufficiency should be interpreted with caution [24]
BMP9	Moderate (inversely correlated with sepsis severity, 50%–80%) [26]	Unknown (more clinical validation needed) [26]	Decreased serum concentrations were significantly associated with 28 day mortality and could be used as a prognostic marker; potential host-oriented therapeutic targets [25]	The mechanism is not clear, the detection method is not popular, and there is a lack of large-scale prospective research validation [26]
Endothelin	High (a sensitive indicator of early vascular endothelial injury, > 80%) [27]	Moderate (50%–80%, clinical exclusion of other vascular diseases) [28]	Early diagnosis of sepsis-related vascular endothelial dysfunction; levels are positively correlated with disease severity and prognosis [25]	Complex detection techniques (e.g., LC-MS) and high cost of dynamic monitoring; nonsepsis-specific (e.g., cardiogenic shock may also be elevated) [29]
Vasoactive peptides	Moderate (associated with hemodynamic disturbances, 50%–80%) [30]	Low (< 50%, diverse mechanisms, insufficient specificity) [31]	Adjunct evaluation of vascular dystonia (e.g., vasopressin deficiency) in septic shock to guide vasoactive medications [27]	It contains a variety of peptides (such as endothelin and bradykinin), the detection system is not uniform, and the clinical application is still in the research stage [30]

## Supporting Information

The Supporting Information provides additional details to support the main text, including the following:

Detailed search syntax used in PubMed, including specific keyword combinations and strategies to retrieve sepsis biomarker and precision medicine studies.

Exclusion criteria and reasons for eliminating nonrelevant studies (e.g., case reports and incomplete data).

Quality assessment tools based on PRISMA guidelines, outlining criteria for evaluating study design, sample size, and statistical validity.

These Supporting Information enhance the transparency and reproducibility of the research, providing readers with comprehensive data and methodological details to support the findings and conclusions of the manuscript.
