# Supplementary material for: Research Progress of Biomarkers for Sepsis and Precision Medicine
Source: Emerg Med Int. 2025 Jul 7;2025:4585495. doi: 10.1155/emmi/4585495 (PMC12259334; doi:10.1155/emmi/4585495)
Supplement: Supporting Information — Additional supporting information can be found online in the Supporting Information section. [file 4585495.f1.docx]

**The PubMed platform was utilized to retrieve data from the Medline database via the following systematic search strategy (October 2019–October 2024). The primary search combined Boolean operators and keyword matching in title/abstract fields to ensure specificity:**

- **For biomarkers:
  ("biomarker"[Title/Abstract] AND "sepsis"[Title/Abstract]) OR ("CRP"[Title/Abstract] AND "sepsis"[Title/Abstract]) OR ("procalcitonin"[Title/Abstract] AND "sepsis"[Title/Abstract])**
- **For precision medicine:
  ("precision medicine"[Title/Abstract] AND "sepsis"[Title/Abstract]) OR ("subphenotype"[Title/Abstract] AND "sepsis"[Title/Abstract])**
- **No MeSH terms were used to avoid under-sampling emerging topics (e.g., novel biomarkers like BMP9 or endothelin), though this approach may limit comprehensiveness.**

**Studies were excluded if they:**

**(1) did not focus on sepsis biomarkers or precision medicine;**

**(2) were non-original research (e.g., reviews, editorials);**

**(3) lacked complete data on biomarker measurements or precision medicine interventions. The PRISMA-compliant screening process is illustrated in Figure S1, detailing the transition from 4,801 identified records to the final included studies.**

**Study quality was assessed using PRISMA guidelines, prioritizing:**

**(1) Study design: RCTs were weighted higher than observational studies due to reduced bias potential.**

**(2) Sample size: Studies with <100 participants were flagged for potential underpowering.**

**(3) Biomarker validation: Only studies using validated assays (e.g., ELISA for PCT) were included.**

**(4) Statistical reporting: Studies lacking clear effect sizes (e.g., odds ratios, 95% CI) were excluded.**

**In this study, the sensitivity and specificity grading of biomarkers are based on the commonly used numerical range of clinical diagnostic tests and the consensus of the existing literature, which are defined as follows:**

1. **Sensitivity**

**High：>80%（Strong ability to identify true positive cases）**

**Moderate：50%-80%（Moderate recognition ability, with some false negatives）**

**Low：<50%（Poor recognition ability and easy missed diagnosis）**

1. **Specificity**

**High：>80%（It has a strong ability to exclude true negative cases）**

**Moderate：50%-80%（Moderate exclusion with some false positives）**

**Low：<50%（The exclusion ability is poor, and it is easy to misdiagnose）**

**Grading basis description**

**This criterion refers to the usual thresholds for diagnostic test accuracy studies（as《Diagnostic Test Accuracy: Fundamentals and Practice》）,Combined with the evaluation conclusions of biomarkers in the classic literature in the field of sepsis (such as the results of meta-analysis of traditional markers such as CRP and PCT).）.**

**For emerging markers that lack well-defined values (e.g., BMP9, vascular endothelin), grading is based on comparison with known markers in the same class and qualitative evaluation of available clinical study data.**
